# Management of Acute Traumatic Spinal Cord Injury: A Review of the Literature

**DOI:** 10.3389/fsurg.2021.698736

**Published:** 2021-12-13

**Authors:** Timothy Y. Wang, Christine Park, Hanci Zhang, Shervin Rahimpour, Kelly R. Murphy, C. Rory Goodwin, Isaac O. Karikari, Khoi D. Than, Christopher I. Shaffrey, Norah Foster, Muhammad M. Abd-El-Barr

**Affiliations:** ^1^Department of Neurological Surgery, Duke University Medical Center, Durham, NC, United States; ^2^Department of Orthopedic Surgery, Duke University Medical Center, Durham, NC, United States; ^3^Premier Orthopedics, Centerville, OH, United States

**Keywords:** traumatic spinal cord injury, review, complications, surgery, management

## Abstract

Traumatic spinal cord injury (TSCI) is a debilitating disease that poses significant functional and economic burden on both the individual and societal levels. Prognosis is dependent on the extent of the spinal injury and the severity of neurological dysfunction. If not treated rapidly, patients with TSCI can suffer further secondary damage and experience escalating disability and complications. It is important to quickly assess the patient to identify the location and severity of injury to make a decision to pursue a surgical and/or conservative management. However, there are many conditions that factor into the management of TSCI patients, ranging from the initial presentation of the patient to long-term care for optimal recovery. Here, we provide a comprehensive review of the etiologies of spinal cord injury and the complications that may arise, and present an algorithm to aid in the management of TSCI.

## Introduction

Despite recent advances in understanding the pathogenesis and treatment of traumatic spinal cord injury (TSCI), it remains a devastating event, often resulting in severe and permanent disabilities. According to the American Association of Neurological Surgeons (AANS), ~450,000 persons living in the United States are permanently disabled due to TSCI with ~11,000 new cases each year (1). TSCI often occurs in young, healthy adults, which results in decades of lost productivity and quality adjusted life years (QALY). While medically complex and life-disrupting, TSCI also poses a significant economic burden. It has been estimated that annual medical treatment costs range from $30,770 to $62,653 per year (2). The authors present an overview of the pathophysiology and presentation of acute TSCI and provide an algorithm that can help guide evaluation and management of patients with TSCI.

## Pathophysiology and Mechanism of Injury

The pathophysiology of TSCI involves a sequential order of events categorized into two phases. The primary injury results from compression of the spinal cord by pressure from bony fragments, blood products, soft tissue, and/or foreign objects. Vasogenic shock follows the inciting event and results in spinal cord ischemia. Soon after the onset of the first insult, there is a release of cytokines and vasoactive proteins that cause inflammation and cord edema, worsening the ischemia and promoting cell death (3). Dying neurons release free radicals and fail to reuptake glutamate neurotransmitters, resulting in oxidative damage and excitotoxicity (4, 5).

## Clinical Presentation

Patients with TSCI can present with either complete or incomplete injury. Traditionally, a complete injury means no voluntary motor or conscious sensory function below a certain level of injury. This definition, however, is often inadequate in certain clinical scenarios. For example, patients can have areas of preserved function below the level of injury, termed zone of partial preservation. Similarly, a patient may also have asymmetric lateral preservation. As a result, an injury is only classified as complete if there is no motor or sensory function in the anal and perineal region representing the lowest sacral segments (S4-5). The American Spinal Injury Association (ASIA) scoring system is used to universally describe the severity of a patient's spinal cord injury (Table 1) (6). We review the ASIA categories below.

**Table 1:** The American Spinal Injury Association (ASIA) scoring system.

**Description of clinical presentation**	**Grade**
Complete: no preservation of function below level of injury, and no sacral sparing (S4-S5)	A
Incomplete: sensory but not motor function is preserved below the neurological level with sacral sparing	B
Incomplete: motor function is preserved below the neurological level, and more than half of key muscles below the neurological level have a muscle grade <3	C
Incomplete: motor function is preserved below the neurological level, and at least half of key muscles below the neurological level have a muscle grade of 3 or more	D
Normal: motor and sensory function are normal	E

### Complete Injury

In a complete injury (ASIA grade A), examination reveals a no motor function, no sensory function below neurological level of injury, and no sacral sparing. In the acute setting, reflexes are absent (including bulbocavernosus) and male patients may have priapism. Urinary retention and bladder distension also occur. Patients with cervical or high thoracic complete cord injuries can suffer variable sympathetic dysfunction including hypotension and bradycardia.

### Incomplete Injury

Incomplete injuries (ASIA grades B through D) preserve voluntary anal contraction, have non-zero perineal sensory scores and often preserved bulbocavernosus reflex. Furthermore, there are various degrees of motor function and sensation caudal to the level of injury. Sensation is often preserved to a greater extent than motor function.

Central cord syndrome is an incomplete pattern of injury. The classic mechanism is a hyperextension injury which is exacerbated by pre-existing cervical spondylosis and/or central canal stenosis. The exam is characterized by disproportionally greater motor impairment in the upper (especially distal upper) vs. lower extremities, often with bladder dysfunction and variable degree of caudal sensory loss. Anatomically, this distribution can be explained by the medial somatotopy of the arms in the long spinal tracts.

Anterior cord syndrome is another incomplete pattern of injury. This is characterized by injury affecting the anterior two-thirds of the spinal cord often secondary to anterior spinal artery injury from either vascular occlusion (embolic stroke) or ligation. Direct mechanical injury to the anterior cord can also occur from disc/bone fragment retropulsion, often with flexion as the causative mechanism. This region includes corticospinal tracts, spinothalamic tracts and descending autonomic tracts, while preserving the posterior column. As a result, patients experience complete motor paralysis and loss of pain and temperature, although have preservation of tactile position and vibration.

Also known as lateral hemisection or hemicord syndrome, Brown-Sequard injuries involve unilateral damage to the dorsal column, corticospinal tract and spinothalamic tract. Patients experience ipsilateral weakness, loss of vibration and proprioception, and contralateral loss of pain and temperature sensation beginning approximately two spinal levels below the injured level. This unique pattern of contralateral sensation loss is due to the decussation of spinothalamic fibers that occurs approximately two spinal levels above the level of injury since these fibers have yet to decussate and are thus preserved. Common causes of this syndrome include ballistic and penetrating injuries.

### Spinal Shock

Immediately following spinal cord injury, there may transient loss of complete spinal cord function below the level of injury with largely unremarkable imaging. These injuries are likely secondary to transient loss of potassium within injured cells, its accumulation in the extra-cellular space with gradual normalization as seen with improvement in the clinical exam (7). At the time of initial injury, the spinal cord appears normal, though overtime, hemorrhagic foci develop within the gray matter leading to accumulation of edema and protein aggregates. This eventually results in central necrosis and vacuolization. Unlike SCI, spinal shock is a result of physiologic, rather than anatomic, reflex depression of spinal cord function.

Spinal shock is transient, and its temporal course is measured by the return of spinal reflexes usually starting with the bulbocavernosus reflex (as early as 1 h after injury), followed by the anal cutaneous reflex, and subsequently the plantar reflex (8). The temporal course and order of reflex recovery is under debate, but there is consensus that recovering reflexes indicate that there was a component of spinal shock at time of original injury (9). On the contrary, patients with permanent areflexia with or without replacement by pathological reflexes are more likely to have suffered from mechanical spinal cord injury (10).

Of note, it is important to distinguish between spinal shock and neurogenic shock. Whereas, spinal shock can occur from damage to any region of the spinal cord, neurogenic shock usually occurs with cervical and high thoracic (i.e., above T6) vertebral levels. Hence, the symptoms seen with neurogenic shock are aligned with sympathetic dysfunctions such as hypotension, hypothermia, and bradycardia. The occurrence of the two shocks is not mutually exclusive of one another.

## Evaluation

### In the Field

As per Advanced Trauma and Life support (ATLS) guidelines, airway, breathing, and circulation “A, B, C's” are the first priorities for Emergency Medical Services (EMS) responding to patients with suspected spinal cord injury. Any patient suspected of having spinal cord injury, conscious or unconscious, must be placed in spinal precautions on a backboard with their head rigidly secured with tape or blocks, and their neck placed in a rigid cervical collar (typically a Miami J collar or Aspen collar) (11). The arms must remain secured at the patient's side with spider straps (12). These rigid mechanisms allow for safe transport from the field to the hospital, where clinical and radiographic evaluations can take place (13). There are many advanced braces such as cervicothoracic orthoses (CTO) and thoracolumbosacral orthoses (TLSO) that serve to provide structural support for stable injuries or following surgical stabilization of unstable injuries, but they are typically not factored into the acute management TSCI so we will defer discussion.

### In the Trauma Bay

Patients with spinal cord injury will often have other injuries and have a high likelihood of spinal fractures. Up to 80% of patients with TSCI have been reported to have multiple injuries (14, 15). Conversely, a polytraumatized patient should be assumed to have spinal cord injury until appropriate diagnostic testing has been performed to eliminate the diagnosis (16). Polytrauma management should be performed with the assistance of the emergency department providers, as well as the trauma surgery team to reassess the patient's airway, breathing, and circulation before additional diagnostic studies. If possible, the patient's MAP should be maintained between 85 and 90, although other injuries and possible bleeding must also be taken into consideration (16, 17). In particular, cranial hemorrhages or great vessel injuries such as aortic dissections may require reductions in systolic blood pressure. If blood pressure augmentation will result in worsening hemorrhagic shock, then spinal perfusion blood pressure goals should be deferred until deemed safe. Once the patient is hemodynamically stable, a trauma series computed tomography (CT) is performed, which should include a CT brain without contrast, CT cervical spine without contrast, and CT chest, abdomen and pelvis with maximum intensity protocol (MIP) with and without contrast from which thoracic and lumbar spinal reformats can be made. Plain radiographs may be used as needed to characterize orthopedic injuries to the extremities, especially important to consider if exam suggestive of extremity pain, weakness or paresthesias. If there is an injury pattern suspicious for head/neck vascular injury, a CT head and neck angiogram is ordered. Specifically, these injuries include atlanto-occipital or atlanto-axial dissociation injuries, cervical spine fractures that extend through the transverse foramen, or rotational injuries such as those resulting in unilateral or bilateral jumped or perched facets.

**Figure 1:**
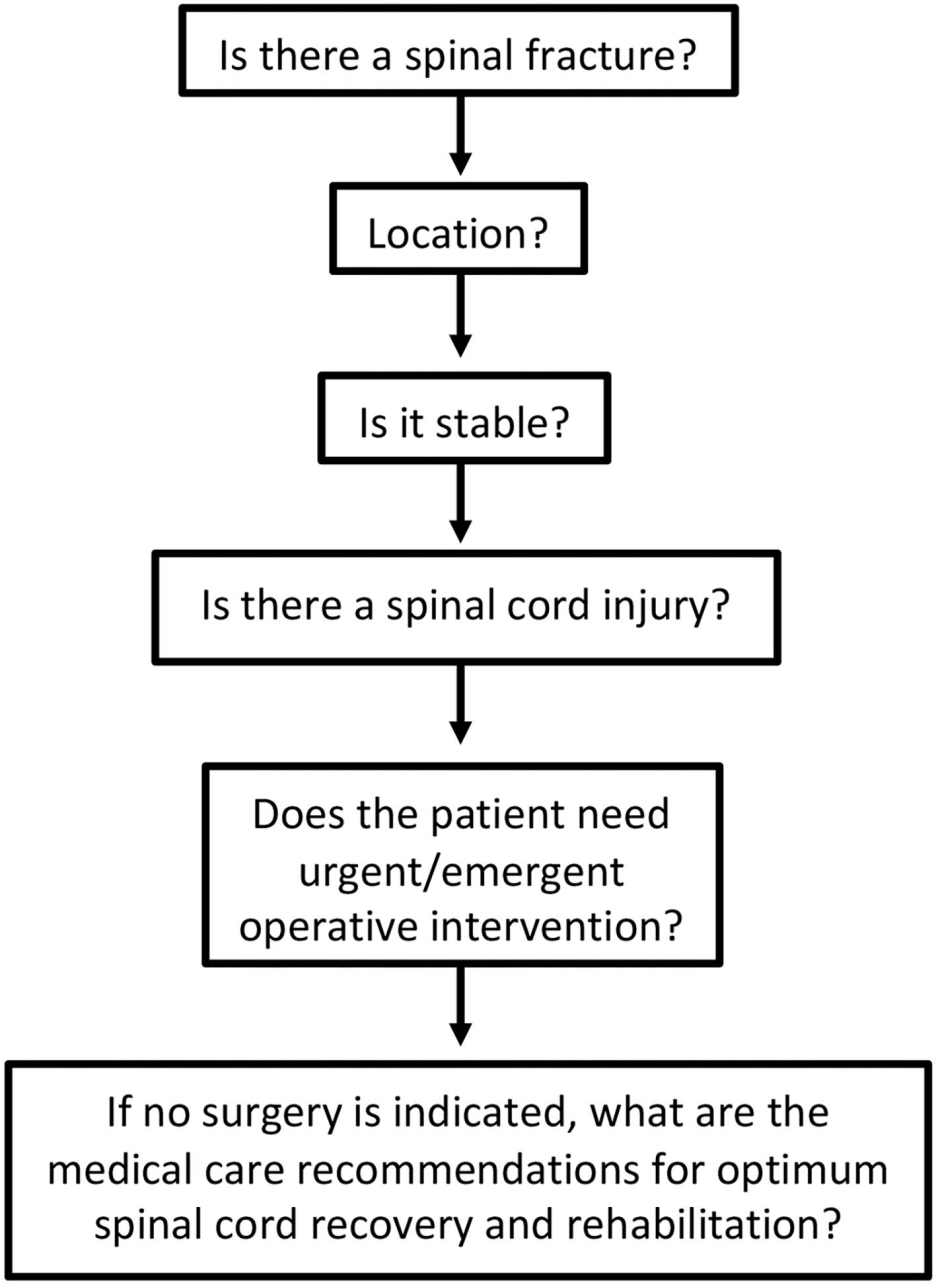
Evaluation process of spinal cord injury.

If a spinal fracture or abnormality is determined on initial imaging, the on-call spine surgery provider should be consulted for further recommendations. For patients with suspected spinal cord injury (weakness or other neurological changes) in absence of spinal fracture, spine service consultation or neurology consultation can be useful to determine diagnosis, as well as provide guidance on the timing of additional diagnostics and management of possible cord injury that may have occurred without the presence of a bony fracture (i.e., central cord syndrome). Evaluation of spinal cord injury can proceed along the pathway shown in Figure 1.

In addition to CT, magnetic resonance imaging (MRI) can also be considered during evaluation. The utility of MRI is several-fold. In cases where no obvious traumatic spinal fracture has occurred, it has the ability to demonstrate spinal cord edema or occult ligamentous injury. Additionally, MRI has superior fluid definition, and thus compressive hematomas are best visualized using this modality. Additionally, MRI can help surgeons determine ligamentous integrity, presence or absence of traumatic disc herniation, as well as degree of spinal stenosis. These aspects make MRI helpful in formulating care plans for patients with suspected or confirmed spinal cord injury.

The major drawbacks to MRI include scanner availability, acquisition time, and the possibility of retained metal and/or unknown medical history to clear patient for safe entry into the magnet, which can be common in TSCI with variable levels of consciousness from pathological or iatrogenic insults. In many cases, balancing the delays involved in MRI acquisition with the need for urgent intervention has challenged the utility of MRI in acute management of SCI. In a 2011 review of 113 articles discussing MRI utility in the management of spinal cord injury, Bozzo et al. suggested 3 recommendations: (1) based on weak evidence, MRI should be used in all patients with spinal cord injury to help guide management, (2) based on moderate evidence, MRI should be used in all patients with spinal cord injury to help guide prognostication, and (3) based on moderate evidence, all spinal MRIs should include a sagittal T2 sequence to identify mechanical spinal cord injury or transection, compression or edema and to assist with prognostication (18). Thus, for patients with acute spinal cord injury, the authors agree that every effort should be made to obtain an MRI of the relevant spinal section to assist with operative planning, diagnostics, and prognostication, but this should not delay emergent operative intervention where the area of injury is clear with an actively declining neurological exam, to reduce an unstable fracture, or otherwise in a hemodynamically unstable patient.

## Medical Management

### Cardiovascular Complications

In the acute stage following SCI, cardiovascular complications require prompt medical attention to prevent neurologic compromise and morbidity. Specifically, the disruption of the sympathetic nervous system that commonly occurs in patients with severe spinal cord injuries at T6 or higher can lead to autonomic dysreflexia including hypotension (both supine and orthostatic) and cardiac arrhythmias (usually bradycardia). Current management guidelines support treatment of hypotension (as defined by systolic blood pressure <90 mmHg) with fluid resuscitation and vasopressor as needed. Further augmentation can be considered following hemodynamic stability in order to achieve the spinal perfusion goals of MAPs >85 or 90, which we discuss further below.

#### Recommendation

A pressor (i.e., Dopamine) with sympathetic, chronotropic and inotropic cardiac support is recommended unless contraindicated (19). Following dopamine, the second-line agent that is recommended is norepinephrine due to it also providing a combination of alpha- and beta- adrenergic support. Vasoactive agents that induce reflex bradycardia, such as phenylephrine, should be avoided in injuries above ~T6 since the body may not be able to appropriately mount a sympathetic response to bradycardia. However, phenylephrine can be considered appropriate in lower thoracic injuries given vasodilation pathology (20).

### Blood Pressure Augmentation

As mentioned above, the occurrence of hypotension is associated with worse outcomes (21). Laboratory evidence suggests hypotension results in poor spinal cord perfusion and contributes to secondary injury and worse neurological outcomes (22). There is prospective and retrospective evidence (Table 2) that mean arterial pressure (MAP) augmentation in the acute phase may improve neurologic outcomes. The American Association of Neurological Surgeons (AANS) currently recommends that MAP be maintained above 85–90 mmHg for 7 days post-injury (30). This practice has yet to be validated with high-level evidence and remains an ongoing area of study. The seven-day recommendation takes root in the Vale et al. landmark studies of the 1990's, which derived this window from animal SCI models. More recent studies have applied a shorter duration of 5 days, and Hawryluk et al. have described data suggesting that higher MAP values best correlate with neurologic recovery in the first 2–3 days post injury, with weaker correlation over the remainder of the 7-day window (26–29, 31, 32). Given that aggressive pursuit of MAP goals can carry vasopressor-related risks, the specifics of this protocol bear further clarification with high level investigation.

**Table 2:** Summary of association between mean arterial pressure and neurological outcome in acute spinal cord injury.

**References**	**Description**	**Evidence class**	**Conclusion**
Vale et al. (23)	Prospective assessment of 77 SCI patients treated with aggressive hemodynamic support, MAP >85 (no control group) × 7 days	III	Improved outcome with aggressive medical care, distinct from potential benefit from surgery at 1-year follow-up
Levi et al. (24)	50 patients treated with aggressive medical treatment, MAP > 90 × 7 days	III	Improved outcome with aggressive hemodynamic support at 6-week follow-up
Levi et al. (25)	103 SCI patient (50 incomplete, 52 complete injuries), hemodynamic support, MAP > 85	III	Improved neurological outcome (no difference between early and late surgery group)
Tator et al. (26)	144 SCI patients managed with aggressive hemodynamic support	III	Improved neurological outcome, less mortality and earlier transfer from ICU care
Zach et al. (27)	Prospective assessment of 117 SCI patients with aggressive pressure support	III	Improved neurological outcome with aggressive medical treatment and blood pressure management
Dakson et al. (28)	Retrospective review of MAP pressure trends in 94 SCI cases	III	Higher rates of neurologic recovery in patients who maintained MAP > 85 mmHg consistently over course of 5 days
Hawryluk et al. (29)	Retrospective review of MAP pressure trends by minute in 100 SCI cases	III	Higher average MAP values correlated with improved recovery in first 2–3 days for those who had 5 days of support

*ICU, intensive care unit; MAP, mean arterial pressure; SCI, spinal cord injury*.

#### Recommendation

Based on the currently available literature, the authors suggest the maintenance of MAP above 85–90 mmHg after SCI has the potential for improving neurological recovery by improving spinal cord perfusion and thereby reduce secondary injury. The optimum duration of MAP goals is at the discretion of the surgeon and critical care teams with an understanding that most literature demonstrating its efficacy applied these goals for 5–7 days with earlier cessation of blood pressure augmentation if no neurological improvement is seen after the first 72 h, or if there is evidence of complete cord transection. This recommendation was also independently reached from a literature review performed by Saadeh et al. (20).

### Respiratory Complications

Pulmonary complications following SCI is common, owing to diaphragm innervation from C3 to C5 level and thoracic accessory muscle innervation from the thoracic roots. In fact, the most immediate cause of early death is due to cardiopulmonary arrest. Patients with high cervical cord injury often require ventilatory support and continued mechanical ventilation with tracheostomy. Lower cervical and thoracic SCI may need temporary ventilatory support with eventual wean from mechanical ventilation to independent breathing. Due to decreased cough strength, patients remain at risk for developing chronic pulmonary complications including pneumonias and atelectasis.

#### Recommendation

Vaccination against influenza and pneumococcus for all patients with respiratory impairment due to SCI is recommended (33, 34). Aggressive daily chest physiotherapy with bed percussion, deep suctioning, and respiratory recruitment maneuvers are beneficial in promoting airway compliance and clearance.

### Role of Steroids

Methylprednisolone is suggested to improve neurological outcomes in patients with acute, non-penetrating TSCI. The evidence is limited, and its use heavily debated. In animal experiments, glucocorticoids have been shown to reduce edema, prevent intracellular potassium depletion, and ultimately improve neurological outcome (35). In humans, most research has been generated by the potential benefit of methylprednisolone. The most widely recognized studies include National Acute Spinal Cord Injury Study (NASCIS) I (36), II (37), and III (38) published between 1990 and 1998. NASCIS I compared high-dose to low-dose methylprednisolone (high-dose: 1,000 mg loading dose followed by 1,000 mg daily, low-dose: 100 mg loading dose followed by 100 mg dose daily). At 6-month follow-up, no difference was found in motor or sensory outcomes. Wound infections and mortality were however more frequent in the high-dose group compared to the low-dose group (*P* = 0.01).

In NASCIS II, methylprednisolone and naloxone administration were studied in 487 randomized patients with acute SCI. Methylprednisolone was administered in an initial loading dose of 30 mg/kg followed by 5.4 mg/kg/h for 23 h. In a *post-hoc* analysis (Class III evidence), the authors reported a mean improvement of five points in the motor score and four points in sensory score at 6-months with sustained improvement in motor score at 1-year, but not in light touch and pinprick. Given this *post-hoc* analysis, 291 patients randomized after 8-h were excluded from the study and therefore conclusions were based on 66 methylprednisolone treated patients vs. 69 controls. Regarding complications, there was a 1.5-times higher incidence of gastrointestinal hemorrhage, 2-times higher incidence of wound infection, and 3 times higher incidence of pulmonary embolus in the steroid group compared to controls. Although none of these findings were statistically significant, the study was not properly powered for this. NASCIS III study compared 24-h, 48-h, tirilazad + 48 h of methylprednisolone infusion without a control group given the findings that were concluded from the prior study. Again, within all *ad-hoc* comparisons, there were no significant differences; however, in *post-hoc* analyses motor function was improved in patients who received 48-h infusion compared to 24-h if initiated within 3- to 8-h of injury. This improvement of five motor points was significant at 6-weeks and again at 6-months, but statistically questionable at 1-year (*P* = 0.53).

While the prior studies demonstrated an increased infection risk with a 48-h regimen of high-dose methylprednisolone sodium succinate (MPSS), lower complication rates were observed with a shorter, 24-h course of high-dose MPSS (30 mg/kg bolus followed by a 5.4 mg/kg/h infusion for 23 h) while still providing long-term neurological benefits (39). Similarly, a 2012 Cochrane review summarizing 6 large-scale studies on MPSS in acute SCI did indeed find an overall increase in ASIA motor scores when MPSS was used, but only if the initial dose was given within 8-h of injury (40). Still, the 2013 AANS/CNS spine section guidelines for acute SCI management do not recommend MPSS in the treatment of SCI (41). Subsequent AOSpine 2017 guidelines from an international expert panel's systematic review subsequently demonstrated only modest improvement in motor scores if MPSS was given within 8-h of injury, with no significant increase in complication rates between 24-h MPSS infusion and the no-steroid control group (42).

#### Recommendation

The authors do not believe there is consistent or compelling evidence to suggest high-dose methylprednisolone administration improves outcomes in TSCI based on current literature (Table 3). In fact, there is evidence to suggest it is associated with increased complications including infection, respiratory compromise, GI hemorrhage and death. Therefore, we do not recommend that methylprednisolone be used routinely in spinal cord injury patients.

**Table 3:** Summary of association between methylprednisolone use and neurological outcome in acute spinal cord injury.

**References**	**Description**	**Evidence class**	**Conclusions**
Matsumoto et al. (31)	Prospective, randomized, double blind I study in 46 SCI patients for the purpose of comparing medical complications	I	Methylprednisolone patients had higher incidence of complications (56.5 vs. 34.8%, NS) Respiratory complications (P = 0.009) and GI bleed (P = 0.036) were significantly higher in MP patients
Pointillart et al. (32)	Multicenter, prospective, randomized I clinical trial of 106 SCI patients treated with MP, nimodipine, MP + nimodipine, or no pharmacological agent	I	No difference in neurological outcome between groups at 1-year (small sample size) Infection, GI bleed, and hyperglycemia higher in MP patients
Bracken et al. (38)	NASCIS III	I (Reported positive results III)	*Post-hoc* analyses showed improved ASIA motor scores at 6-weeks and 6-months in 48 MP patients compared to 24 MP
Bracken et al. (38)	NASCIS II: 1-year follow up	I (Reported positive results III)	*Post-hoc* analyses showed improvement in motor but not sensory scores at 1 year in patients given MP within 8 h of injury
Bracken et al. (43)	NASCIS II	I (Reported positive results III)	*Post-hoc* analyses showed improvement in motor and sensory scores at 6-months in patients given MP within 8 h of SCI

*ASIA, American Spinal Injury Association; GI, gastrointestinal; MP, methylprednisolone; NASCIS, National Acute Spinal Cord Injury Study; NS, not significant; SCI, spinal cord injury*.

## Surgical Management

### Candidacy

While the primary injury to the spinal cord is irreversible, the secondary injury is ongoing and can be attenuated if addressed in a timely manner (44). Numerous studies have shown that prompt decompression and/or fixation enhance restoration of neurological function (45–52). Surgery should be considered in patients who are likely to benefit from decompression, mechanical stabilization, fracture reduction, and deformity correction. Such interventions have the potential to eliminate the source of further secondary injury and promote patient recovery.

### Timing of Surgery

Mechanism, type of injury, severity of other bodily injuries, and clinical exam are crucial in determining appropriate timing of surgery following SCI. Given the inherent heterogeneity of injury, unbiased studies are difficult to conduct and limit evidence-based consensus in the field. For example, in cases of acute cord injury where the pathology of disease is a herniated disc causing ischemia secondary to anterior spinal artery (ASA) compression with evidence of exam deficits, timely surgery is imperative. However, outcomes are variable between complete and incomplete spinal cord injuries. In one meta-analysis of 30 studies, 13 demonstrated improved outcomes in early decompression, while 14 reported no statistical difference and 2 reported increased neurologic deterioration (53). In a recent randomized multicenter trial (Surgical Timing in Acute Spinal Cord Injury Study [STASCIS]), 313 patients with acute cervical SCI were randomized to early surgery (<24 h) or late surgery. Of the 222 patients with follow-up available at 6-months post injury, 19.8% of patients undergoing early surgery showed a >2 grade improvement in AIS compared to 8.8% in the late decompression group. Despite 30% of patients being lost to follow-up, this is the largest randomized controlled clinical trial addressing timing of surgery in SCI since 2000 (47). The only subsequent other randomized controlled trial by Rahimi-Movaghar et al. did not find a statistically significant improvement in motor strength at 12-months postoperatively between early (within 24 h) vs. delayed (> 24 h) intervention, but these results are limited by small sample size (*n* = 35) (54). In a 2010 survey of spine surgeons, the majority (>80 percent of 971 respondents) reported a preference to decompress the spine within 24 h of SCI. Shorter time intervals (within 6–12 h) are preferred by the majority of surgeons for certain lesions, including incomplete cervical SCI. STASCIS along with other studies outlined below (Table 4) are part of a growing body of evidence that favor early decompression following SCI. A prospective cohort study of 888 patients by Dvorak et al. found improved motor recovery in patients who underwent decompression and stabilization within 24 h (59). Furthermore, there is evidence that early intervention within 72 h of injury is associated with fewer overall complications during admission such as pneumonia, pressure ulcers, and UTI (58). If patients are shown to have an actively declining spinal exam in the presence of mass effect or mass lesion, emergent operative intervention is indicated (61). Otherwise, patients with stable neurological exams who undergo decompression within 24 h have the potential for improved outcomes compared to late decompression. For example, the recently published study by Badhiwala et al. showed that patients who had decompressive surgery within 24 h experienced greater recovery, higher total motor and sensory scores, and had better ASIA grades at 1 year after surgery compared to those who had surgery later than 24 h post-SCI (60).

**Table 4:** Summary of relationship between timing of surgery and clinical outcomes in patients with spinal cord injury.

**References**	**N**	**Location**	**Timing**	**Intervention**	**Results**
Ng et al. (55)	26	Cervical	± 8 h	Laminectomy	Patients with surgical decompression within 8 h showed significantly shorter overall hospital and intensive care unit stay and had fewer systemic complications and improved neurological outcomes
Cengiz et al. (56)	27	Thoracic/Lumbar	± 8 h	Decompression and stabilization	Patients with surgical decompression within 8 h showed significantly shorter overall hospital stays and better neurological outcomes
Fehlings et al. (47)	313	Cervical	± 24 h	Decompression and stabilization	Patients with decompression within 24 h had ≥2 ASIA scores at 6-month follow-up than those receiving delayed surgery (≥24 h)
Wilson et al. (57)	84	Cervical/Thoracic/ Lumbar	± 24 h	Decompression and stabilization	Patients with decompression surgery <24 h post-injury had greater ASIA motor recovery than those with surgery ≥24 h post-SCI
Bourassa-Moreau et al. (58)	431	Cervical/Thoracic/ Lumbar	<24 h vs. 24–72 h	Decompression and stabilization	Intervention within 72 h post-injury predicted lower complication rates such as pneumonia, UTI during hospitalization
Rahimi-Movghar et al. (54)	35	Thoracic/Lumbar	± 24 h	Decompression and stabilization	No significant difference in motor recovery at 12-months postoperatively for patients undergoing surgery <24 h of injury compared with those undergoing surgery between 24 and 72 h, limited by small sample size (*n* = 16 early; *n* = 19 delayed surgery patients)
Dvorak et al. (59)	888	Cervical/Thoracic/ Lumbar	± 24 h	Decompression and stabilization	Surgical intervention <24 h of injury associated with increased ASIA motor recovery and in ASIA A, B patients, association with significantly shorter LOS
Badhiwala et al. (60)	1,548	Cervical/Thoracic/ Lumbar	± 24 h	Decompression and stabilization	Patients who had early decompression experienced greater recovery, higher total motor and sensory scores, and had better ASIA grades at 1 year after surgery

*ASIA, American Spinal Injury Association; LOS, length of study; SCI, spinal cord injury; STASCIS, Surgical Timing in Acute Spinal Cord Injury Study; UTI, urinary tract infection*.

#### Recommendation

While there is no definite Class I proof for this cohort, the authors recommend that urgent decompression within 24 h be performed for patients with stable neurological exams and emergent decompression should be pursued for patients with actively declining neurological exams.

## Rehabilitation

### Diet

Spinal cord injury results in metabolic changes owing to decreases in mobility and energy consumption. As a result, frequent dietary changes are required, especially if a patient's neurological function begins to recover. Doing so can avoid unwanted comorbidities including diabetes, excessive spinal stress, and lipid disorders that may be secondary to weight gain.

#### Recommendation

It is recommended that a nutritionist be involved in designing dietary plans for each patient with spinal cord injury, which should be updated in the acute, subacute, and chronic recovery period to ensure that the necessary modifications to caloric needs are addressed. Early feeding within 72 h, if safe and recommended based on expert opinion for critically ill patients, but this has not been shown to affect neurological outcomes, length of stay, or incidence of complications (62). This is particularly important for patients with high cervical spinal cord injury or patients who cannot otherwise tolerate standard oral intake and may rely on tube feedings as their primary source for nutrition (63).

### Bowel Management

Changes in the gastrointestinal (GI) system following spinal cord injury are important to address as part of comprehensive spinal cord injury rehabilitation. While gastric digestion and nutrient absorption remains relatively stable following acute SCI, other functions such as bowel evacuation undergo significant changes and, if mismanaged, have the potential to cause morbid complications (64). In cases of high-cervical injury, there is impairment in esophageal sphincter control, which may increase rates of gastroesophageal reflux disease (GERD) and lead to delayed gastric emptying with high post-feed residuals. The incidence of gallbladder disease also increases for patients with SCI above the T10 level as a result of impaired gastric mobility (65). In both upper and lower cord injuries, colonic stasis precipitates ileus and there is loss of voluntary control of the external anal sphincter in complete injuries. Bowel management thus becomes complex and nuanced. It is suggested that patients with spinal cord injury should increase their dietary fluid and fiber intake to increase the water content of their stool to decrease gastric transit time, though data supporting this practice is limited (66, 67). Stool softeners, such as docusate and polyethylene glycol, should be administered on a scheduled basis. These can be combined with bowel stimulants such as senna glycoside. Bulk formers such as psyllium or methylcellulose expand the stool volume within the bowel to stimulate peristalsis. Bowel stimulant usage patterns are much more varied. Cisapride (serotonin-4 receptors) is a less common agent that has been associated with cardiac arrhythmias and is thus rarely used. Metoclopramide usage has recently increased, but it is important to recognize that this upper GI stimulant is contraindicated in patients with bowel obstruction. Especially important for complete SCI is incorporation of contact irritants such as rectal stimulation, suppositories, and enemas to maintain bowel regularity.

#### Recommendation

We suggest patients with spinal cord injury be placed on scheduled stool softeners with titration of bulk formers, suppositories, and enemas as needed to achieve at least one bowel movement per every-other-day. In patients with complete cord injury, rectal stimulation should be performed daily, with adjustments by frequency of bowel movements (68–70).

### Venous Thromboembolism Prevention and Treatment

Thromboprophylaxis is standard of care for patients in the acute stage following SCI. Evidence based guidelines from the American College of Chest Physicians support the use of mechanical and thromboprophylaxis for at least the initial 2 weeks following injury (71). Specifically, thromboprophylaxis with low-molecular weight heparin (LMWH) within 72 h of injury should be initiated, along with intermittent pneumatic compression devices (72). Although there are no trials specifically addressing the duration of DVT prophylaxis, the majority of DVT events following SCI occur within the first 8 weeks of injury. Thus, the Spinal Cord Consortium recommends at least 8 weeks of pharmacologic DVT prophylaxis following acute SCI (73, 74). The routine use of IVC filters is not recommended, as multiple studies in the trauma literature have not established its efficacy in reducing overall or PE-related mortality with substantial associated costs. IVC filters are indicated in cases where thromboprophylaxis has failed or there is contraindication to blood thinning (i.e., active bleeding) (75).

#### Recommendation

For acute patients, LMWH thromboprophylaxis with pneumatic compression device should be started within 72 h of injury for a duration of at least 8 weeks.

### Urogenital Complications

Unlike cauda equina syndrome, patients with spinal cord injury have a functional conus disconnected from other normal neurological input. As a result, patients with spinal cord injury will display involuntary reflux detrusor contractions during bladder filling (similar to detrusor over activity) with reflex contraction of the distal sphincter resulting in detrusor-sphincter dyssynergia. In these patients, bladder compliance is normal, but they have absent or greatly reduced awareness of bladder filling. As a result, urinary retention is to be expected in patients with spinal cord injury, which increases the risk of urinary tract infections, hydronephrosis, renal stones and post-renal kidney failure (76). In the setting of acute injury, a urinary catheter is necessary to track accurate fluid balance. It should be noted that, if able, a post-void bladder scan should be done prior to the placement of a catheter to help understand initial presence of urinary retention. Void trials can be pursued either during hospitalization or in rehabilitation with the trend toward earlier removal of indwelling catheters followed by scheduled catheterizations based on bladder volume to prevent catheter-associated urinary tract infections (CAUTIs) (77). Oftentimes, patients with spinal cord injury will exhibit the permanent loss of independent voiding. Scheduled clean intermittent self-catheterization is most commonly performed, but other options such as external male urinary catheters or suprapubic catheters also exist depending on upper extremity function and/or degree of retention. For patients with some retained sensation and voluntary control, micturition through straining and sacral root stimulation are possible, although these are likely inadequate methods of maintaining a properly decompressed bladder. For patients with bladder spasms, oxybutynin is a viable option at providing patient comfort while also reducing incontinence associated with spasms. Tamsulosin, a well-tolerated alpha-inhibitor, functions to reduce sphincter tone through smooth muscle relaxation to improve bladder emptying (78). However, this should be avoided in the acute injury period, as alpha-inhibitors may inadvertently lower the blood pressure and reduce spinal cord perfusion.

#### Recommendation

We recommend that a urinary catheter be placed following initial bladder scan and removed for void trials when strict fluid balance monitoring is no longer necessary. For patients who fail void trials, patients should be allowed to maximally independently void with assistance of alpha-inhibitors and acetylcholine antagonists, and/or intermittent self-catheterization at scheduled intervals, which decrease in frequency if retained volume decreases over time (79). For patients who are incapable of performing any of the above, suprapubic catheterization remains a suboptimal method for bladder decompression.

### Mental Health Following SCI

Sudden and unexpected losses of mobility, bowel or bladder function, sexual function, and independence have significant effects on mental health and perceived quality-of-life. In a study of 443 patients with SCI, Migliorini et al. found high incidences of depression (37%), anxiety (30%), and post-traumatic stress disorder (8.4%) with up to 48.5% of patients experiencing some form of mental health disorder (80). Additionally, SCI patients have lower perceived quality-of-life and higher levels of distress than members of the general population (81).

#### Recommendation

While there are no systematic approaches to mental health in this patient population, providers should remain aware of the increased prevalence of psychiatric disorders and have a low threshold to offer the appropriate mental health resources as needed.

### Mobility and Disposition

Perhaps the most complex element of post-acute care for patients suffering from spinal cord injury is in the area of physical rehabilitation. This is especially true for patients with complete injury, who have suddenly lost movement in their lower and possibly upper extremities. The acute loss of mobility is not only psychologically disturbing, but it has downstream effects including increased risk of diabetes, obesity, and pressure ulcers (82). It is recommended that patients with acute spinal cord injury be engaged with physical therapy as early as post-injury day one, with the target goal being >20 min of maximum-tolerated aerobic activity per day (83). Additional aerobic activity though swimming, walking, and wheelchair mobility (as best tolerated) are positively associated with better quality of life outcomes, although these have not been compared to no-activity in randomized controlled trials (84). The more activity that is tolerated, the better the outcome with respect to all aspects of chronic disease (85, 86). Eventually, discharge to spinal cord injury rehabilitation is imperative, as these centers have physical therapy and resources that specialize in spinal cord injury patients, and evidence suggests that appropriate disposition and rehabilitation has the ability to add years to the lives of patients with spinal cord injury (87).

#### Recommendation

Early engagement with physical therapy specialists, with at least once-daily sessions for at least 20 min of activity, is recommended to encourage aerobic conditioning and muscle retention. Eventual disposition should be focused on entering an outpatient rehabilitation center specialized in managing spinal cord injury patients.

## Other Investigational Therapeutic Options

### Spinal Cord Cooling

In an effort to prevent biochemical injury that ensues following an initial insult, spinal cooling has been considered. In a case series analyzing 20 patients, Hansebout et al. used a combination of surgical decompression, steroid administration, and regional hypothermia with a “cord cooling” device in treatment of complete cord injuries (88). Of the 20 patients treated, 13 had improvement from initial ASIA A impairment. Currently in the literature, there are only similarly small clinical studies and case reports that describe the impact of systemic hypothermia in treating SCI (89, 90). Controlled, randomized clinical trials are needed to establish its benefit. Due to limited evidence and conflicting evidence in brain cooling studies, the authors do not recommend use of cooling as standard of care in TSCI.

### Role of GM-1

Gangliosides are complex acidic glycolipids that form a major component of the cell membrane. While little is known about the functions of neuronal gangliosides, there is experimental evidence that they induce regeneration and sprouting of neurons and restore neuronal function. In animal studies, these compounds have been shown to stimulate the growth and regeneration of damaged nervous tissue. While encouraging, translational studies have failed to show a similar benefit in humans (91). At present the NASCIS II protocol, involving GM-1 ganglioside (300-mg loading dose followed by 100 mg/d for 56 days) initiated after the administration of methylprednisolone, is not recommended in the treatment of adult patients with SCI.

### Electrical Stimulation

Electrical stimulation (ES) is a relatively new treatment strategy that has shown promise to improve neurologic function in cases of *chronic* spinal cord injury in animals and humans (92–95). The mechanisms by which electrical stimulation works to improve neurological function are not entirely known but it is theorized that ES may help facilitate neurological recovery by stimulating neuronal outgrowth due to electrical fields (96, 97) and also by increasing excitability of neuronal networks below the lesion (94, 98). Much of the work regarding epidural stimulation has revolved around stimulating in the lumbar region to help improve lower extremity function (93, 99, 100). Recently, Krucoff et al. has successfully shown improvement in motor function in a patient with L1 complete spinal cord injury with epidural stimulation at T12-L1 (101).

A naturally occurring electrical field exists in the wall of the early neural tube and is required for guiding cranial-to-caudal nervous system development. The trophic and tropic effects have therefore been investigated in *in vitro* and *in vivo* models of seal lamprey, rodent, and canine TSCI and have been found to improve functional outcomes (102–106). A novel human oscillating field stimulator has been proven safe in a phase I trial (96, 107). Further research is needed to suggest use of spinal cord stimulation (SCS) in acute TSCI.

### Autologous Macrophages

Recent investigations have focused on immune system-mediated repair of injury. In a randomized controlled trial, Lammertse et al. enrolled participants with complete TSCI between C5 motor and T11 neurological levels within 14 days of injury to receive either a 2:1 ratio autologous incubated macrophages or control injection. Treatment group participants underwent macrophage injection into the caudal boundary of the SCI with primary outcome measure of ASIA A-B or better at ≥6 months. The study found no difference in outcomes between these two groups (108). Research is ongoing to establish a role for immunomodulation in SCI.

### Lumbar Drainage

Ischemia of the spinal cord is an important factor in secondary damage after SCI. In fact, in the setting of thoracoabdominal aortic aneurysm surgery it can be the cause of paralysis and cardiac surgeons frequently utilize cerebrospinal fluid (CSF) drainage during such repairs (109, 110). Lowering intrathecal pressure (ITP) by draining CSF theoretically promotes perfusion, but this mechanism has not been extensively studied in the setting of TSCI. Conceptually, if increasing MAPs is thought to increase spinal cord perfusion pressure (SCPP) and be beneficial for neurological outcomes, then decreasing ITP should achieve similar outcome (SCPP = MAP – ITP). In a pig model of SCI, 15 pigs were studied comparing SCI alone, SCI with MAP goals, and SCI with MAP goals + CSF drainage. This study demonstrated that the combination of MAP elevation and CSF drainage significantly and sustainably improved blood flow and spinal cord perfusion pressure (111). In a human study by Kwon et al., lumbar drainage was undertaken in a randomized fashion in 22 patients within 48 h of injury (112). Lumbar drainage after 72 h was not associated with significant adverse events, but the study lacked statistical power to support neurological benefit. Newer studies have demonstrated better efficacy and safety profiles, though this theory remains unsubstantiated (113, 114). A similar concept of reducing intrathecal pressure can also be applied to patients undergoing patch duraplasty during time of surgical decompression. Phang et al. performed a trial evaluating intraspinal pressure and fluid dynamics between spinal cord injury patients undergoing laminectomy or laminectomy with patch duraplasty and found that intrathecal pressure and spinal cord perfusion pressure were improved in patients undergoing duraplasty. While ASIA impairment, bladder function and bowel function were improved in the duraplasty group, this did not reach statistical significance, suggesting that larger trials with long-term follow-up are needed to better evaluate the clinical implications of duraplasty (115).

### Riluzole

A critical component of the SCI pathophysiology is the intrinsic neural cellular response to mechanical injury. Uncontrolled activation of voltage-gated sodium channels has been hypothesized as necessary in the cytotoxic response leading to secondary injury in spinal cord trauma. Riluzole is a sodium channel blocking anticonvulsant that has been clinically validated for human applications in treating amyotrophic lateral sclerosis (ALS). It has also been postulated as a potential tool to reduce neuronal apoptosis in the acute phase following TSCI. Preclinical trials have shown promise in laboratory-induced TSCI models (116). Currently, Riluzole in Acute Spinal Cord Injury Study (RISCIS) is an ongoing multi-center controlled trial that forms the basis of a Phase II clinical investigation to determine its efficacy in the acute treatment of SCI, with a dose recommendation of 100 mg immediately in the first 24 h following diagnosis of injury, followed by 50 mg every 12 h for an additional 13 days (117). Interestingly, a recent study by Fehlings et al. showed that riluzole may not be helpful in helping with patients' functional recovery after surgery for degenerative cervical myelopathy (DCM) (118). The authors await the results of the RISCIS study to make formal recommendations for its routine use.

### Tissue Scaffolding

New trials utilizing biomaterial scaffolds for spinal cord repair have recently emerged. These trials utilize similar biochemical and anatomical principles seen in the repair of the peripheral nervous system. Preliminary work has demonstrated that biomaterial scaffolds synthesized from either a natural or synthetic polymer can concentrate neurotrophic growth factors while promoting axonal regeneration between the two ends of the injured neural tissue. Growing axons may thus reconnect with neurons caudal to the injury site, thus reconstituting the circuitry. To date, this has only been demonstrated in the animal models with limited efficacy seen in human trials (119, 120). A large clinical trial with six enrollment sites (“INSPIRE,” NCT02138110) was initiated in 2014 and aimed to determine the benefit of a poly(lactic-co-glycolic acid)-b-poly(L-lysine) scaffold in subjects with thoracic ASIA A traumatic thoracic spinal cord injury. This study, however, is not actively enrolling patients and its last update was in 2019. Recommendations for tissue scaffolding are pending further investigational and translational research.

## Summary of Recommendations

**Figure 2:**
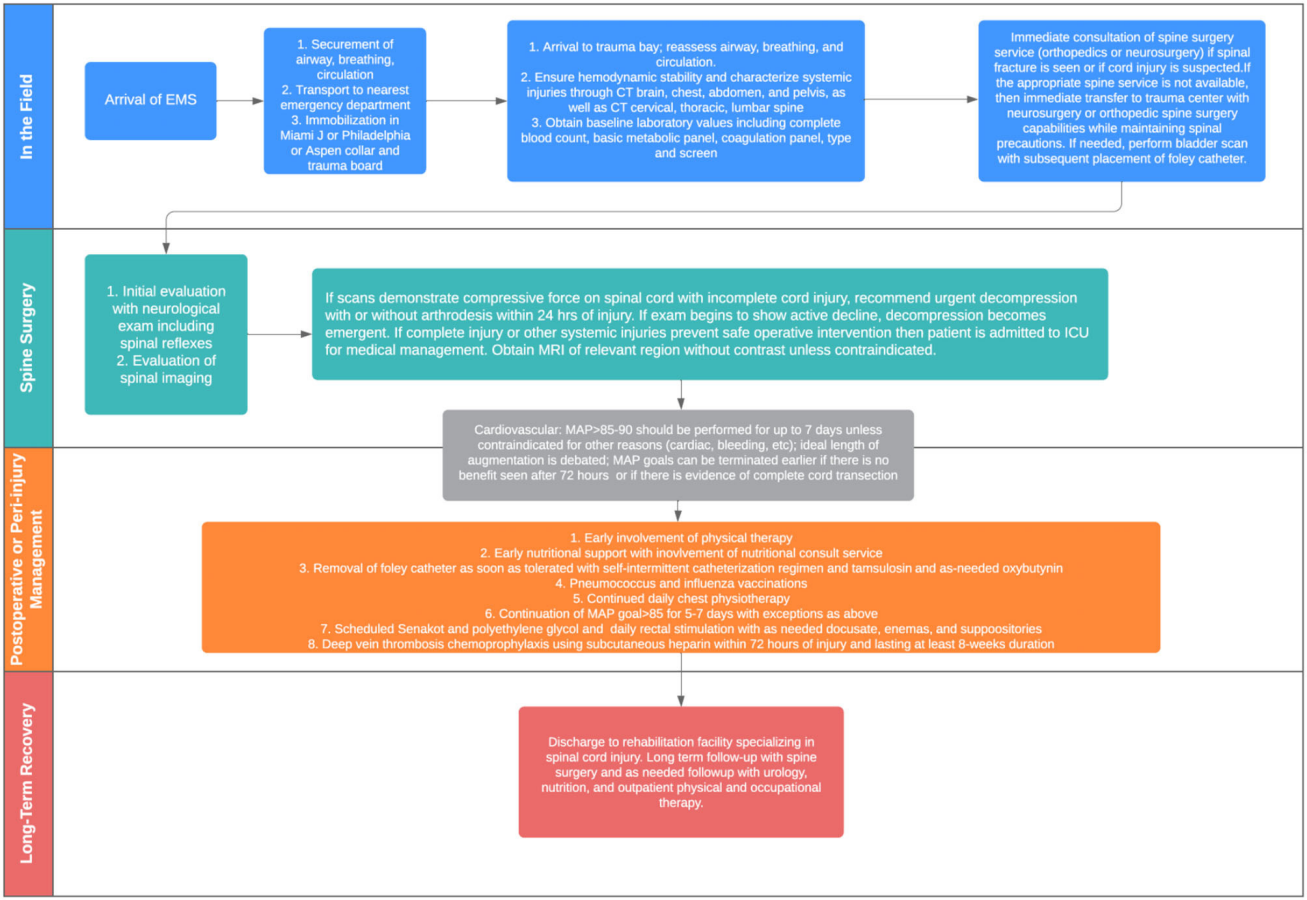
Algorithm for managing acute spinal cord injury.

Figure 2 provides an overview of the algorithm that guides decision-making for treating patients with acute traumatic spinal cord injury at the authors' institution. At the initial evaluation stage, CT total spine imaging should be performed promptly in all cases of suspected SCI, and if at all possible, magnetic resonance imaging is recommended to evaluate suspected cord injury and to help guide surgical management and prognostication. However, emergent surgical intervention should not be delayed for the purposes of an MRI. Surgical treatment, if indicated, can and should be performed within 24 h of injury and is associated with improved neurologic outcome. Recommendations for a complete injury are less clear and treated on a case-by-case basis. Patients with actively declining exams should be considered for emergent decompression.

For medical management, chest physiotherapy and vaccination against pneumonia causing pathogens for all patients with respiratory impairment due to SCI is recommended. Maintenance of MAP 85–90 mm Hg after SCI for 5–7 days is safe and may improve spinal cord perfusion to improve or stabilizeneurological outcome. The threshold goal and the length of augmentation need further definition. Routine administration of methylprednisolone (MP) for the treatment of acute SCI is not recommended.

Rehabilitation is essential to reduce the risk of development of further complications and to help regain function in patients suffering from spinal cord injury. Early involvement of nutritional specialists can facilitate appropriate adjustments in fat, protein, and carbohydrate intake to prevent nutritional deficiency and/or obesity. Patients should be started on aggressive scheduled bowel regimen beginning immediately and should include use of osmotic stool agents and bulk-formers. Early void trials with conversion to intermittent catheterization as needed can reduce the risk of urinary complications and can be facilitated with alpha-blockers such as tamsulosin and acetylcholine antagonists such as oxybutynin. Frequent screening and early referral to mental health resources is recommended. Finally, early mobility and eventual disposition to rehabilitation centers with expertise in spinal cord injury can extend and improve quality of life.

## Conclusion

Acute traumatic spinal cord injury can leave a devastating impact on the physical and mental well-being of patients. For emergent cases in which the patients present with severe neurological deficits, it is essential to provide prompt and specialized treatment. After initial evaluation involving appropriate physical examinations and imaging, those who are deemed necessary to undergo surgical evaluation. For both surgical and non-surgical candidates, medical management should be undertaken as needed to optimally stabilize the patients. Additional considerations such as diet, urinary and bowel activity, mental health, and mobility are also important in minimizing disability and ultimately restoring functional capabilities.

## Author Contributions

TW performed the literature search. TW and CP prepared and critically revised the original draft. All authors contributed to the study conception and design and reviewed the final draft.

## Conflict of Interest

The authors declare that the research was conducted in the absence of any commercial or financial relationships that could be construed as a potential conflict of interest.

## Publisher's Note

All claims expressed in this article are solely those of the authors and do not necessarily represent those of their affiliated organizations, or those of the publisher, the editors and the reviewers. Any product that may be evaluated in this article, or claim that may be made by its manufacturer, is not guaranteed or endorsed by the publisher.
